# Assessing the quality and reliability of the Amazon Mechanical Turk (MTurk) data in 2024

**DOI:** 10.1098/rsos.250361

**Published:** 2025-07-16

**Authors:** Hagar Shimoni, Vadim Axelrod

**Affiliations:** ^1^ The Gonda Multidisciplinary Brain Research Center, Bar-Ilan University, Ramat Gan, Israel

**Keywords:** Amazon Mechanical Turk, MTurk, online experiments, data quality, reliability, master workers, approval rate, attentional check items

## Abstract

Amazon Mechanical Turk (MTurk) has been one of the most popular platforms for online research in psychology and the social sciences in general. While concerns about MTurk data quality have been raised, the platform continues to be widely used. The question is whether the MTurk platform is suitable for research and, if so, whether it is used optimally. We conducted a systematic investigation of MTurk data quality and reliability, including main and replication experiments, with more than 1300 participants subdivided into three cohorts: (i) workers (i.e. participants on the MTurk platform) with master requirement (i.e. high-performing workers selected by MTurk), (ii) workers without master requirement, and (iii) workers without master requirement, but with a 95% or above approval rate. We found that master workers almost never missed attentional checks, exhibited high reliability and showed no tendency towards straightlining, therefore, these workers are recommended, especially when the naivety of participants is not a strong prerequisite and no large sample size is required. In contrast, the workers without restrictions or with a 95% or above approval-rate threshold missed many attentional checks, exhibited low reliability and showed a tendency towards straightlining, raising serious concerns about the suitability of these workers for research.

## 1. Introduction

Online experiments have become a widely used research method in the behavioural sciences [1,2] while Amazon Mechanical Turk (MTurk) has been one of the largest and most popular platforms. Originally, research using the MTurk platform was met with great enthusiasm [3,4], but in recent years, concerns have arisen regarding the quality of MTurk data [5–8]. Critically, as we illustrate below through a survey of papers published in 2024 in the psychology field, MTurk continues to be widely used in research. So, can the data obtained using MTurk be trusted? Notably, as the data quality of MTurk is constantly changing [5,6,9,10], the conclusions and recommendations made even several years ago, let alone 10 years ago, might no longer be adequate. All this underscores that verifying the current quality of MTurk data—the goal of the present research—is both essential and timely.

### 1.1. Strategies for using qualified workers

**Figure 1.**
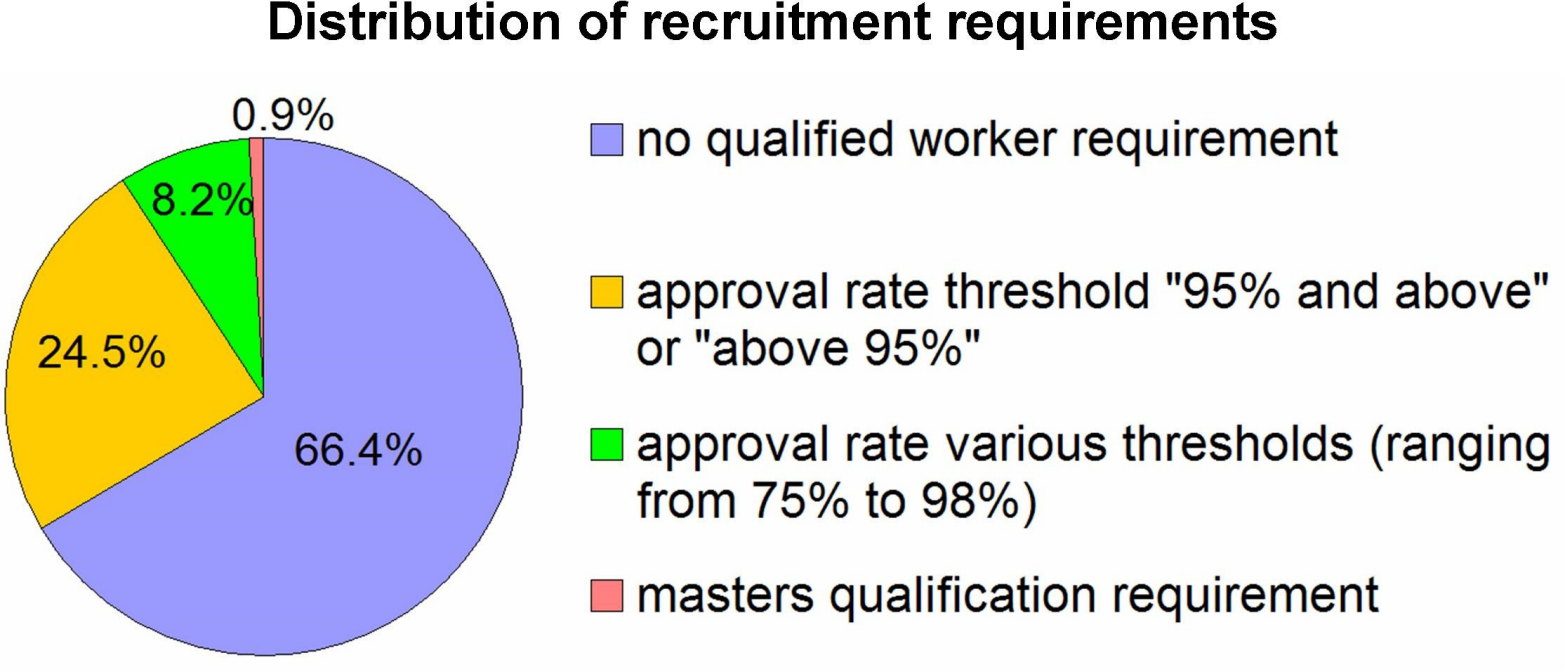
Distribution of recruitment requirements: the survey of psychology papers published in 2024 that used the MTurk platform. The search was conducted in the Web of Science database for papers published in 2024 in the ‘Psychology’ research area using the search term ‘mechanical turk’ or ‘MTurk’ in the abstract, title, author keywords or keywords plus fields. A total of 110 papers were identified. Note, that two-thirds of studies did not require qualified workers, approximately a quarter of the studies used an approval threshold of ‘95% and above’ or ‘above 95%’, and less than 1% (i.e. only one study) required a master qualification.

One of the principal approaches to ensuring data quality has been to limit the recruitment of participants to qualified workers [11–14]. MTurk proposes two options for doing that. The first is to specify a minimal approval rate for participants’ previous tasks. This requirement can also be complemented by requiring a minimal number of completed tasks. The second option is to recruit master workers (i.e. high-performing workers selected by MTurk based on its internal criteria). To conduct a representative estimation of the extent to which MTurk has been currently used in research and how frequently different options for selecting qualified participants have been used, we searched the Web of Science database for papers published in 2024 in the ‘Psychology’ research area using the search term ‘Mechanical Turk’ or ‘MTurk’ in the abstract, title, author keywords or keywords plus fields. We identified 110 empirical papers that used the MTurk platform. Note that these include only papers in the psychology research field, and even within this field, the number represents a lower bound estimate; some studies using the MTurk platform may not have mentioned it in the abstract, title or keywords field. In figure 1, we show the distribution of recruitment requirements. We can see that two-thirds of studies did not require qualified workers while the most commonly used approval thresholds were ‘95% and above’ and ‘above 95%’, accounting for almost a quarter of all published studies. Less than 1% of studies (i.e. only one study) required a master’s qualification. Not shown in the chart, among all studies, 8.1% also required a minimum number of tasks that workers had participated in (most commonly, 100 tasks). How optimal has the strategy used in these studies been?

Selecting participants based on the minimal approval rate, particularly using a 95% threshold, was highly recommended by Peer *et al*. more than a decade ago [12]. But to what extent the use of approval-rate criteria for improving data quality remains beneficial today is unclear. That is, on one side, relatively recent review papers [15,16] still recommended selecting participants based on an approval-rate criterion of 95%. Empirically, the benefit of using a 95% approval-rate criterion was also shown [17,18], although the quality of data obtained from workers with a 95% approval-rate threshold, at least in the case of Peer *et al*.’s study [18], was controversial. On the other hand, Robinson *et al*. [19] found that almost all MTurk workers recruited without restrictions had an approval rate of at least 95% (for similar conclusions, see also an influential monograph of Litman and Robinson [20]). According to this view, selecting participants based on this criterion is effectively equivalent to using unqualified workers. Given that the approval rate remains a widely used option today and given that the data quality of MTurk is changing, it is essential to investigate whether today approval-rate filtering is a meaningful and useful strategy for achieving high-quality data.

In contrast to the approval-rate option and in line with our survey, the ‘Master Requirement’ option has been used only rarely. To some extent, this is surprising, given that this category of workers, which is closely monitored by MTurk, might contain high-performing workers. To date, there have been only a few studies that tried to examine the benefit of applying the ‘Master Requirement’. Specifically, Loepp and Kelly [21], using groups of participants with and without master qualifications, were able to replicate the results of several classic experiments (e.g. [22]); but their study did not directly (e.g. statistically) compare the quality of data between the two groups. In another study using questionnaires [23], the authors found comparable Cronbach’s α internal consistency scores for groups of participants with and without the Master Requirement, suggesting no benefit to using the Master Requirement criterion. Notably, the aforementioned studies were conducted several years ago, and as MTurk quality changes over time, we do not know whether these conclusions still hold today. It is also noteworthy that comparing the quality of data between two groups using Cronbach’s α internal consistency might be biased, especially when the questionnaires do not include reverse-coded items. Cronbach’s α—a measure of the reliability of a questionnaire—assesses whether different items measure the same concept [24], but here we are interested in measuring the reliability of participants, not of a questionnaire. Critically, when a participant provides similar answers to different items on a questionnaire—a straightlining phenomenon characterizing careless participants in general, and online participants in particular [25,26]—Cronbach’s α for that group of participants might be high and inflated [27–33]. Accordingly, a group of participants with a higher tendency for straightlining might show higher Cronbach’s α internal consistency. To this end, a more reliable measure of participants’ reliability might be to calculate correlations across repeated items within participants, when the same or rephrased questionnaire is administered twice [34,35]. Consider, for example, an extreme scenario: for a questionnaire with a [1−7] scale, half of the participants randomly select 1 and 2, while the other half randomly select 6 and 7. Cronbach’s α for this group would be close to 1, but the correlation across repeated items within participants would be close to 0 (i.e. the item answered as 1 on the first repetition might be answered as 2 on the second and vice versa). Note that the method of correlating responses across repeated items within participants [34,35] is conceptually similar to a widely used approach of validating data quality by checking whether the same (or similar) responses are given to pairs of items with similar content (e.g. the Variable Response Inconsistency and True Response Inconsistency scales of the Minnesota Multiphasic Personality Inventory (MMPI) [36]).

### 1.2. Attention checks as an approach to ensure data quality

An additional approach to ensuring data quality in online experiments, and MTurk experiments in particular, has been the use of attentional checks (or ‘catch’ trials) [6,37–39]. Exclusion of data from participants who fail attentional checks has been shown in the past to improve data quality [18,40,41], but the exclusion procedure alone does not necessarily guarantee that the obtained data are of sufficiently good quality [10]. Notably, in a typical experiment, it is difficult or even impossible for a researcher to evaluate the quality of the remaining data after discarding participants who fail attentional checks. To address this, a dedicated experiment with a special experimental design, such as retesting the same group of MTurk participants a second time (e.g. [42]), might be needed. In light of concerns regarding MTurk data quality, and given the continuing use of MTurk in research (see §1.1), it is essential to verify whether the application of ‘catch’ trials today achieves the desired effect.

### 1.3. The current study

The goal of the present study was to systematically investigate the quality and reliability of MTurk data by examining three cohorts of participants: workers without master qualification requirement and without approval-rate requirement (‘No requirements’), workers without master qualification requirement, but with a requirement of 95% or above approval rate (‘95% approval’) and workers with master qualification requirement and without approval-rate requirement (‘Master workers’). We used the Mobile Phone Involvement Questionnaire (MPIQ), consisting of eight items [43], in the main experiment, and the first 10 items of the Boredom Proneness Scale (BPS) [44] in the replication experiment. Our questionnaire included both the original items and closely rephrased versions of them, resulting in a total of 16 and 20 items in the main and replication experiments, respectively. In addition, our experiment included three types of attentional checks of increasing difficulty (i.e. arithmetic ‘catch’ trials, common-sense ‘catch’ trials formulated intuitively and a common-sense ‘catch’ trial formulated contradictorily), permitting us to examine, on the one hand, the quality of the data based on participants’ errors in different attentional checks and, on the other hand, reliability of the participants according to the answers to attentional checks. The reliability of the participants was estimated using within-subject correlation across original and rephrased items [34].

## 2. Methods

### 2.1. Participants

The study was conducted using the Amazon Mechanical Turk (https://www.MTurk.com/) online platform, with data collected in May–September 2024. Participants gave their informed consent by selecting a checkbox. The obtained data were anonymous. The study received approval from the local ethics committee of Bar-Ilan University (ISU202106001), and all phases of the study were carried out in strict compliance with this approval. In total, 1331 participants took part in the main and replication experiments. The participants were from the United States and spoke English as their mother tongue. The participants were paid $2 (main experiment) and $2.5 (replication experiment). In each experiment, there were three cohorts of participants defined using the MTurk set-up requirements: (i) the participants without master requirement or approval-rate requirement (referred to hereafter as ‘No requirements’), (ii) the participants without master requirement, but with approval rate of at least 95% requirement (referred to hereafter as ‘95% approval’), and (iii) the participants with master requirement and no approval-rate requirement (referred to hereafter as ‘Master workers’). In addition, to verify the approval rate of the Master workers, we recruited a separate group of participants with the Master requirement and no approval-rate requirement. The demographic information of the participants is presented in table 1. The sample size was determined before conducting the main experiment in the following way. Our design included three criteria to exclude participants based on the incorrect answers to attentional catch questions of different types (see more details below). In our analysis, we compared the reliability of the three cohorts (i.e. ‘No requirements’, ‘95% approval’ and ‘Master workers’) for each of the attentional catch question’s exclusion criteria (see more details below). Based on our preliminary pilot: (i) applying the most stringent exclusion criterion (i.e. ‘arithmetic and all common-sense catch trials’) resulted in exclusion of approximately 80–85% of participants in the ‘No requirements’ and ‘95% approval’ cohorts, but almost no exclusion of participants in the ‘Master workers’ cohort; and (ii) a comparison of reliability across three cohorts of participants for the most stringent exclusion criterion (i.e. ‘arithmetic and all common-sense catch trials’) resulted in an effect size of approximately *η*^2^ = 0.11 (one-way ANOVA). To achieve this effect size with the power of 0.95 required approximately 45 participants (G* Power program: [45]). Accordingly, the total number of participants had to be approximately 250−300 in the ‘No requirements’ and ‘95% approval’ cohorts and approximately 45−50 in the ‘Master workers’ primary cohort.

**Table 1.** Demographic information of the participants (main and replication experiments). The experiment included three cohorts of participants and a separate group of participants with the Master requirement to verify the participants’ approval rates.

group of participants	demographic information items	main experiment	replication experiment
cohort 1: No requirements	participants number	305	297
	average age (standard deviation)	32.6 (7.5)	32.6 (6.4)
	number of females	107	105
	number of left-handed people	14	13
cohort 2: 95% approval	participants number	296	272
	average age (standard deviation)	34.3 (8.13)	34.3 (7.4)
	number of females	119	75
	number of left-handed people	20	22
cohort 3: Master workers	participants number	59	53
	average age (standard deviation)	45.2 (10.6)	47.6 (11.1)
	number of females	27	26
	number of left-handed people	5	4
approval-rate verification group of Master workers	participants number	24	25
	average age (standard deviation)	43.7 (8.7)	52.3 (11.5)
	number of females	11	16
	number of left-handed people	4	1

### 2.2. Experimental design

We used a combination of two recruitment criteria: participants’ approval rate (on/off) and master qualification (on/off). The approval rate of the participants reflects their performance in previous tasks. We used an approval rate of 95% or above, a recommended and widely used threshold [12,18,46–48], as well as the widely used threshold according to our survey (figure 1). Master participants, defined by master requirement criteria, are high-quality participants selected by MTurk based on their early performance. The MTurk definition of these workers is as follows: ‘Mechanical Turk has built technology which analyzes Worker performance, identifies highperforming Workers, and monitors their performance over time. Workers who have demonstrated excellence across a wide range of tasks are awarded the Masters Qualification. Masters must continue to pass our statistical monitoring to retain the Mechanical Turk Masters Qualification.’ In our experiment (both main and replication), we recruited three cohorts of participants based on the following criteria: (i) ‘No requirements’: neither master qualification requirement nor approval-rate criterion; (ii) ‘95% approval’: there was no master qualification requirement, but a requirement of 95% or above approval rate, (iii) ‘Master workers’: only master qualification requirement, without requirement of approval-rate criterion. In addition, a separate group of participants with the master requirement and no approval-rate requirement was recruited; this group was used to verify the participants’ approval rates. No requirement on the minimum number of completed human intelligence tasks was applied to any group of participants.

After reading the initial greeting message in the MTurk platform, participants had to click on a link to the questionnaire. The questionnaire was in Google Forms. The experiment included items from a widely used existing questionnaire, rephrasing items with the same meaning from this questionnaire and five attentional ‘catch’ items. The design of the main and replication experiments were the same except for the questionnaire used (see below). All items appeared in an arbitrary, pseudo-random order that was different for each participant. In the main experiment, we used a widely used MPIQ with eight items [43] that included questions on mobile phone daily use. None of the items in this questionnaire were reverse-coded. Eight additional items were rephrased versions of the corresponding MPIQ items. By rephrasing the items, we preserved the meaning of the original items as closely as possible. For example, while the original item was ‘I often think about my mobile phone when I am not using it’, the rephrased item was ‘I often ponder my mobile phone even when it’s not in my hands’ (for full list of items, see electronic supplementary material, table S1). The five attentional ‘catch’ items were of three types. First, there were two trivial arithmetic items: ‘Calculate 17 − 12’ and ‘Calculate 37 − 36’. Second, there were two trivial common-sense items formulated intuitively: ‘A runner who completed a marathon in extremely hot weather is likely to be tired’ and ‘A student who failed a very important exam is likely to be upset’. Third, there was a trivial common-sense question formulated contradictorily: ‘A person who has been diagnosed with cancer is likely to feel glad’. The answers to the MPIQ, rephrased MPIQ and the common-sense ‘catch’ questions were given using a Likert scale 1 strongly disagree—7 strongly agree). For uniformity of the answers, the arithmetic ‘catch’ items also had one to seven answers. The questionnaires used were identical for three cohorts of participants (‘No requirements’, ‘95% approval’ and ‘Master workers’). The design of the replication experiment was exactly the same as the main experiment; the only difference was that instead of MPIQ, we used the first 10 items of the widely used BPS [44], which includes questions on a person’s degree of boredom in daily life. Three out of 10 items in this questionnaire were reverse-coded. The full list of original and rephrased items can be found in electronic supplementary material, table S2.

### 2.3. Data analysis

Data analysis was conducted using a custom Matlab code [49]. For each of the three cohorts (i.e. ‘No requirements’, ‘95% approval’ and ‘Master workers’), we created three subgroups of participants based on participants’ responses to attentional catch items. Specifically, in the ‘only arithmetic catches’ subgroup we included the participants who answered both arithmetic items correctly (regardless of the answers to other catch items). We accepted only one correct answer (e.g. for ‘Calculate 17 − 12’, we accepted answer ‘5’). In the ‘arithmetic and intuitive common-sense catches’ subgroup, we included those participants who answered both arithmetic items and both intuitively formulated common-sense items correctly (regardless of the answer to the remaining catch item). We accepted answers 6 and 7 as correct for the intuitively formulated common-sense items (e.g. agreement with ‘A runner who completed a marathon in extremely hot weather is likely to be tired’). Finally, in the ‘arithmetic and all common-sense catches’, we included the participants who answered all catch items correctly. As the correct answer for the contradictorily formulated common-sense item, we accepted only answer ‘1’ (i.e. the lowest level of agreement with ‘A person who has been diagnosed with cancer is likely to feel glad’). Reliability was calculated as the within-participant correlation across items [34,35]. Specifically, for each participant, we correlated the responses between the original and rephrased questionnaire versions. As the data in many cases did not satisfy the assumption of normality, we used non-parametric tests in our analyses (i.e. chi-squared, Kruskal–Wallis, Wilcoxon rank-sum and Spearman correlation).

## 3. Results

### 3.1. Main experiment

First, we compared the demographic characteristics of the three cohorts of participants, and there were no major differences across cohorts with regard to hand dominance (‘No requirements’: 4% with left dominant hand; ‘95% approval’: 8% with left dominant hand; and ‘Master workers’: 4% with left dominant hand; chi-square test for independence: χ^2^(2) = 2.03, *p* = 0.36, Cramer’s V = 0.055). There was also no major difference across cohorts with regard to proportion of genders, although there was a trend for a more balanced dataset with regard to gender in ‘95% approval’ and even more in ‘Master workers’ (‘No requirements’: 35% of females; ‘95% approval’: 40% of females; and ‘Master workers’: 46% of females; chi-square test for independence: χ^2^(2) = 3.18, *p* = 0.2, Cramer’s V = 0.069). Notably, age was different across tested cohorts, with the lowest age in ‘No requirements’ (mean = 32.61, s.d. = 7.5), slightly higher in the ‘95% approval’ (mean = 34.3, s.d. = 8.1) cohort and much higher in the ‘Master workers’ cohort (mean = 45.24, s.d. = 10.56). Statistically, non-parametric Kruskal–Wallis test with the cohort as a factor revealed a very strong main effect of cohort ([H (2) = 82.9, *p* < 0.001, ε^2^ = 0.12). A post hoc non-parametric, two-tailed Wilcoxon rank-sum test revealed a higher age for ‘Master workers’ compared with ‘No requirements’ *(z* = 8.87, *p* < 0.001, *r* = 0.47), ‘Master workers’ compared with ‘95% approval’ *(z* = 7.6, *p* < 0.001, *r* = 0.4) and ‘95% approval’ compared with ‘No requirements’ *(z* = 3.07, *p* = 0.002, *r* = 0.13). In electronic supplementary material S1, we also show the distribution of ages for all three cohorts and can see that in ‘Master workers’ the ages were more evenly distributed.

**Figure 2.**
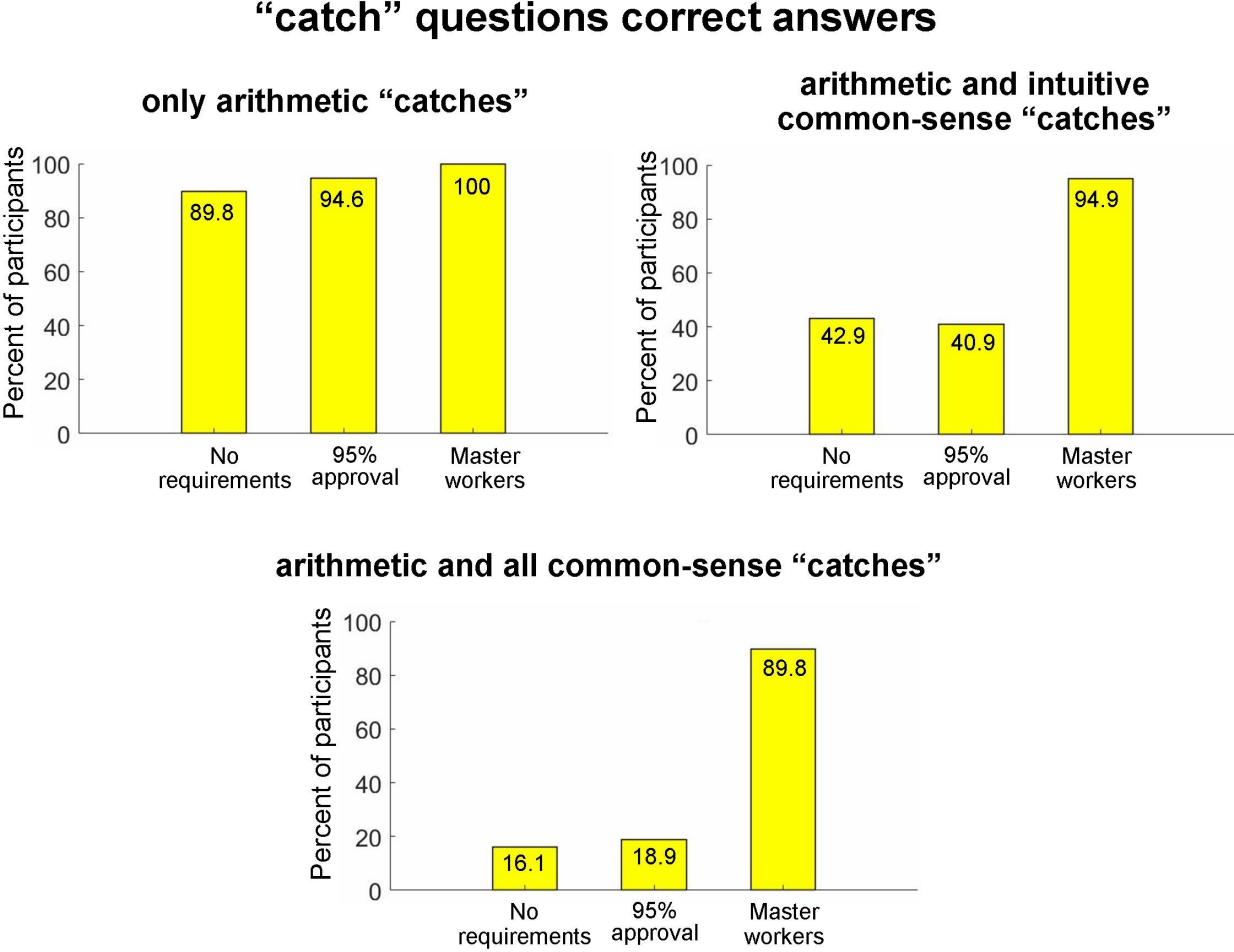
The percentage of participants in each of the three cohorts who correctly answered attentional catch items of three types. Note:. participants in all three cohorts made very few errors in ‘only arithmetic catches’ items (top-left subplot); ‘Master workers’ participants made very few errors on all types of ‘catch’ items (right-most bar in all subplots); in the ‘No requirements’ and ‘95% approval’ cohorts, the percentage of participants who answered all ‘catch’ items correctly was very low (i.e. below 20%; bottom subplot); and there was no major difference between the ‘No requirements’ and ‘95% approval’ cohorts.

Proceeding to the goal of our study, we asked to what extent participants in each of three cohorts answered the attentional ‘catch’ items correctly. Responses to five ‘catch’ items were subdivided into three subgroups: (i) both arithmetic ‘catch’ items were answered correctly, regardless of responses to other ‘catch’ items (‘only arithmetic catches’); (ii) both arithmetic ‘catch’ items and both intuitive common-sense ‘catch’ items were answered correctly, regardless of responses to the contradictory common-sense ‘catch’ item (‘arithmetic and intuitive common-sense catches’); and (iii) all five ‘catch’ items were answered correctly (‘arithmetic and all common-sense catches’). Note that the subgroups contained partly overlapping participants and therefore were not compared statistically. The results for each of three cohorts of participants binned into three catch subgroups are shown in figure 2. We can see that the ‘Master workers’ cohort (right-most bar in all subplots) performed very well: 90% and above of participants answered correctly in all three catch subgroups, including the most stringent ‘catch’ criterion (i.e. ‘arithmetic and all common-sense catches’). In contrast, ‘No requirements’ and ‘95% + approval’ performed well in the ‘only arithmetic catches’ but showed poor performance in the ‘arithmetic and intuitive common-sense catches’ (40% answered correctly) and in the ‘arithmetic and all common-sense catches’ (less than 20% answered correctly). Note that the results of ‘No requirements’ and ‘95% approval’ cohorts were similar, indicating that the 95% + approval restriction might not contribute to data quality.

**Figure 3.**
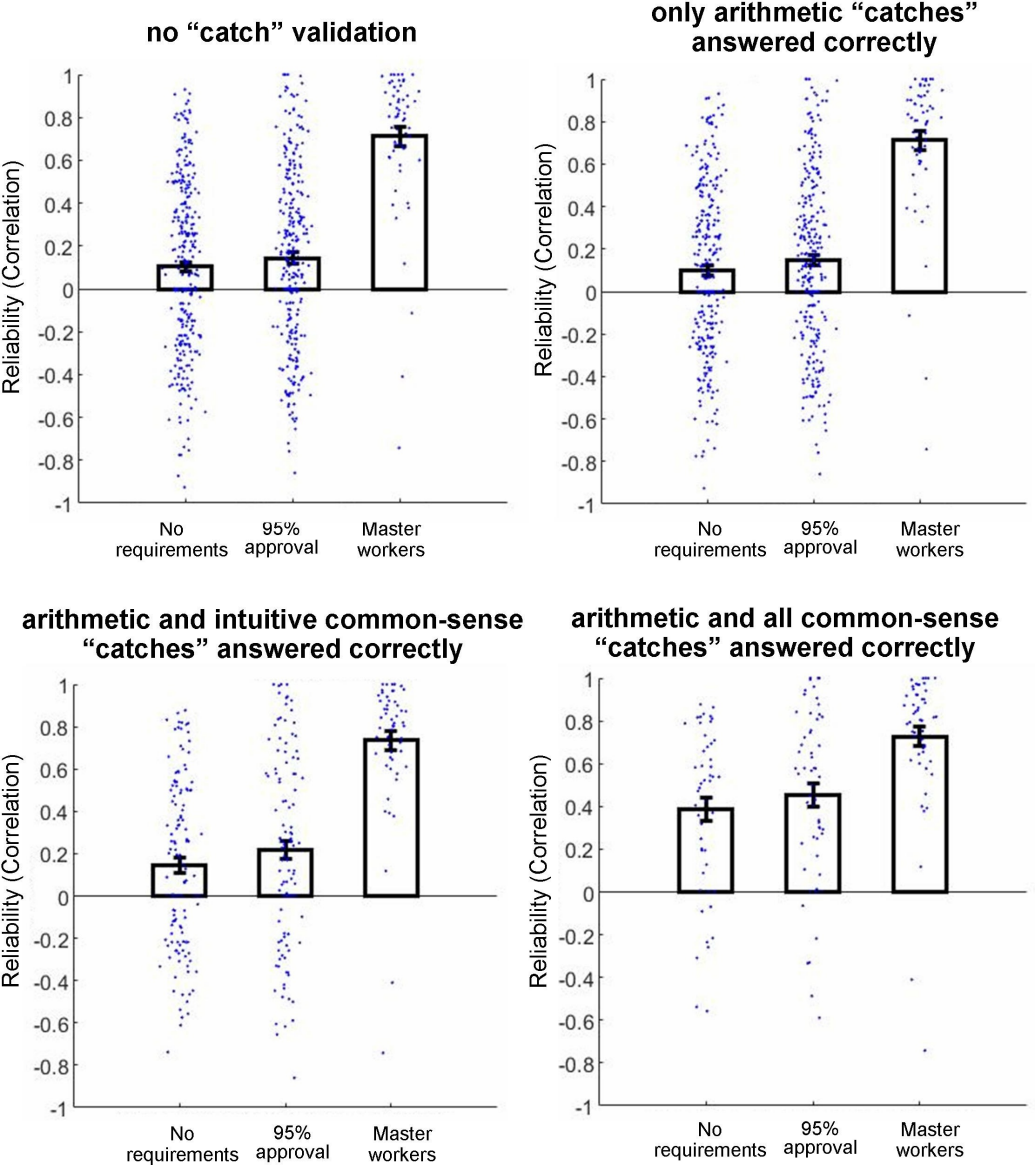
Reliability of three cohorts of participants (‘No requirements’ and ‘95% approval’ and ‘Master workers’) for all data (i.e. no ‘catch’ validation) and three ‘catch’ subgroups. The ‘catch’ subgroups include participants who correctly answered the corresponding attentional ‘catch’ items. Small dots reflect the data of individual participants, and error bars denote the standard error of the mean. Note: the reliability of the ‘Master workers’ cohort was high in all exclusion subgroups; the reliability of two cohorts without master requirement was very low for all the data and the first two ‘catch’ criteria, and intermediate for the most stringent ‘arithmetic and all common-sense catches’ criterion; and there was not much difference in reliability between the ‘No requirements’ and ‘95% approval’ cohorts.

Next, we examined the reliability of the participants’ responses. Before proceeding with the reliability analysis, to be able to calculate correlation we excluded those participants who provided the same or the same except for one answers to all items. In each ‘No requirements’ and ‘95% approval’ cohorts, we excluded 13 participants (4.3% and 4.4%, respectively), and in the ‘Master workers’ cohort, we excluded one participant (1.7%). To calculate reliability, we correlated within participants across eight item scores of two versions of the questionnaire (i.e. the correlation between the original MPIQ items and the rephrased items). In this and in the following, the analysis was conducted separately for all participants (‘no catch validation’) and for each of the ‘catch’ subgroups (the subgroup included only participants who answered the corresponding ‘catch’ items correctly). The results of the reliability analysis are shown in figure 3 (for full statistical results, see table 2), and several aspects warrant emphasis. First, the reliability of the participants without using ‘catch’ trials (figure 3, left-top) and of the subgroup that correctly answered arithmetic ‘catches’ (figure 3, right-top) were almost identical, suggesting the ineffectiveness of arithmetic ‘catch’ trials. Second, the reliability of the ‘Master workers’ cohort was relatively high (i.e. average correlation of approx. 0.7) for all ‘catch’ subgroups. Note that because ‘Master workers’ participants had only a few errors in ‘catch’ trials (figure 2), all ‘catch’ subgroups mostly included the same participants. Third, the reliability of ‘No requirements’ and ‘95% approval’ participants ranged from very low in ‘no catch validation’, ‘only arithmetic catches answered correctly’ and ‘arithmetic and intuitive common-sense catches answered correctly’ subgroups (i.e. the highest average correlation of approx. 0.2) to intermediate in the ‘arithmetic and all common-sense catches’ (i.e. the average correlation below 0.5). Finally, the reliability of ‘95% approval’ participants was only slightly and insignificantly higher than the reliability of ‘No requirements’ participants (see table 2 for full statistical results), suggesting the ineffectiveness of pre-selecting participants based on the 95% approval-rate requirement.

**Table 2.** Statistical results of the reliability analysis according to the attentional catch subgroups. The second column reflects the results of non-parametric Kruskal–Wallis test with the cohort of participants as a factor. The third, fourth and fifth columns reflect the results of post hoc two-tailed Wilcoxon rank-sum comparisons between cohorts. Note: a very strong main effect (i.e. the difference between cohorts) in all ‘catch’ subgroups; much higher reliability in master workers compared with the two cohorts of workers without the master requirement; and no difference in reliability between ‘95% approval’ and ‘No requirements’ cohorts. Statistically significant results are marked in bold.

attentional catch subgroup	Kruskal–Wallis test with cohort as a factor	post hoc two-tailed Wilcoxon rank-sum tests		
		‘Master workers’ versus ‘No requirements**’**	‘Master workers’ versus ‘95% approval**’**	‘95% approval’ versus ‘No requirements**’**
no ‘catch’ validation	H (2) = 92.8, ***p*** **< 0.001**, ε^2^ = 0.14	*z* = 9.47, ***p* < 0.001**, *r* = 0.51	z = 8.83, ***p* < 0.001**, r = 0.48	*z* = 1.07, *p* = 0.28, *r* = 0.04
only arithmetic ‘catches’ answered correctly	H (2) = 91.4, ***p*** **< 0.001**, ε^2^ = 0.15	*z* = 9.37, ***p* < 0.001**, r = 0.52	*z* = 8.73, ***p* < 0.001**, *r* = 0.48	*z* = 1.17, *p* = 0.24, *r* = 0.05
arithmetic and intuitive common-sense ‘catches’ answered correctly	H (2) = 71.3, ***p*** **< 0.001**, ε^2^ = 0.24	*z* = 8.37, ***p* < 0.001**, *r* = 0.62	*z* = 6.8, ***p* < 0.001**, *r* = 0.53	*z* = 1.22, *p* = 0.22, *r* = 0.08
arithmetic and all common-sense ‘catches’ answered correctly	H (2) = 29.8, ***p*** **< 0.001**, ε^2^ = 0.18	*z* = 5.26, ***p* < 0.001**, *r* = 0.52	*z* = 4.04, ***p* < 0.001**, *r* = 0.39	z = 0.96, *p* = 0.33, *r* = 0.09

**Figure 4.**
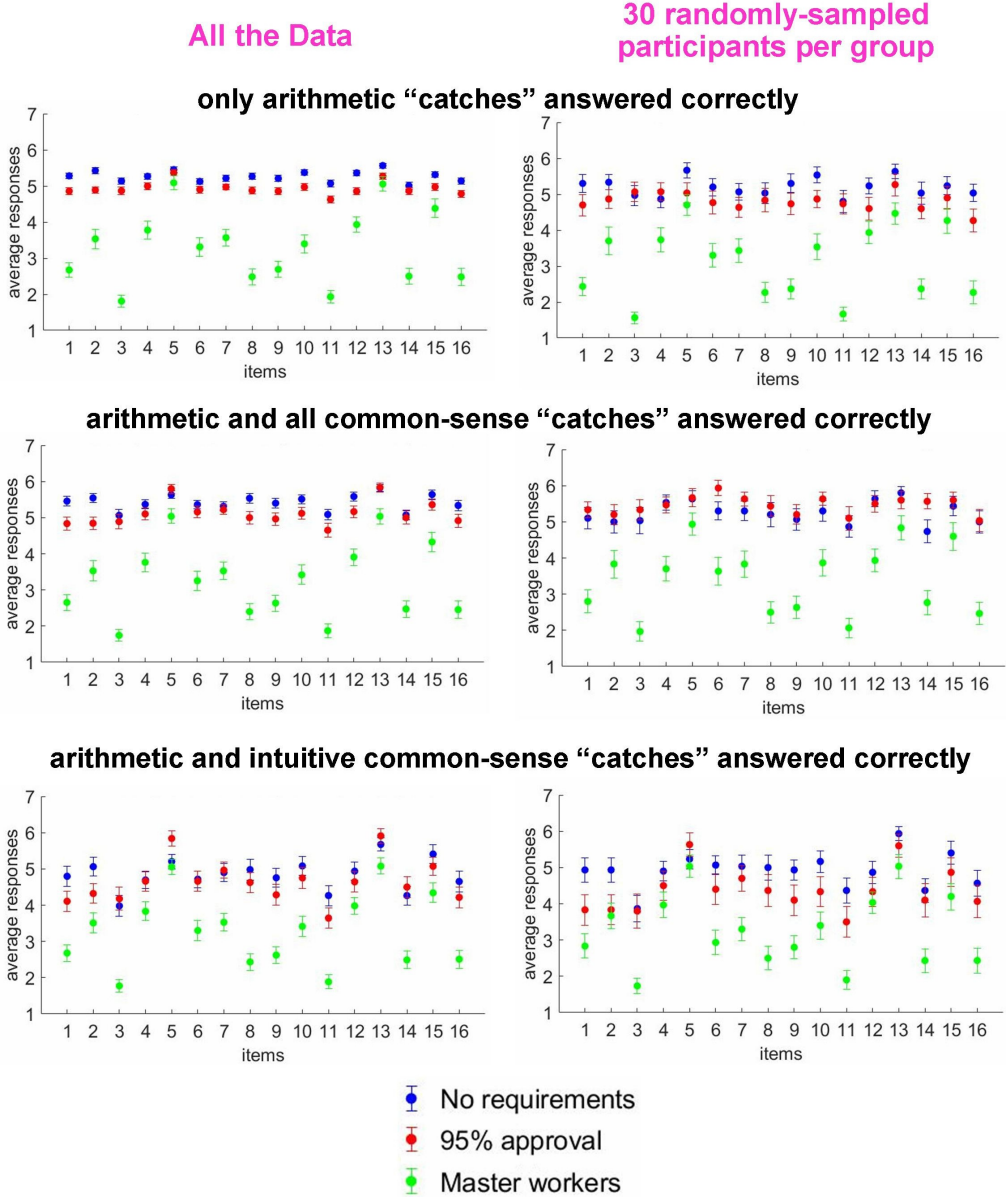
Scores of individual items averaged across participants for three cohorts, binned into three ‘catch’ subgroups. The rows reflect the results of the three ‘catch’ subgroups. The left column: all the data; the right column: the data of 30 random participants. Note that the variability between trials of participants in the ‘No requirements’ (blue dots) and ‘95% approval’ (red dots) cohorts was much lower compared with the ‘Master workers’ cohort (green dots).

Next, to investigate the phenomenon of low reliability of participants without master requirement, for all three cohorts, we plotted scores for each item averaged across participants within the group (figure 4, left column). The scores for ‘No requirements’ and ‘95% approval’ were higher than those for the ‘Master workers’ participants, but this observation by itself cannot explain the lower reliability of the former two subgroups. The more relevant and striking observation was that ‘No requirements’/‘95% approval’ compared with ‘Master workers’ participants, showed much lower variability across items—a reflection of the well-known phenomenon of straightlining [26], a characteristic behaviour of careless responders [34]. Obviously, the less distinctive the responses across items, the lower the signal-to-noise ratio and the lower the reliability of the results. Note that in a more stringent ‘catch’ trial subgroup (‘arithmetic and all common-sense catches’) for the cohorts without master requirement, variability across items increased (figure 4, bottom-right), indicating increased quality of the data. It is noteworthy that in the ‘only arithmetic catches’ and ‘arithmetic and intuitive common-sense catches’ subgroups, the sample size of ‘No requirements’ and ‘95% approval’ participants was much larger than that of the ‘Master workers’ cohort. To rule out the possibility that the cohorts’ different sample sizes might explain differences in variability across trials, we repeated the analysis for all three cohorts and for each of the three ‘catch’ subgroups by randomly sampling 30 participants. As we can see in figure 4, right column, the variability across items for the ‘No requirements’ and ‘95% approval’ participants was still much lower than for the ‘Master workers’ participants. Similar results were obtained when different subsamples of 30 participants were used.

Finally, given the low quality of data from participants in the ‘95% approval’ cohort, we were interested in the approval rate of master workers. To investigate this, we recruited a separate group of 24 participants with the master requirement. These participants were asked to provide their approval ratings. The average approval rating in this dataset was 99.67% (s.d. 0.38%).

### 3.2. Replication experiment

To replicate our results and ensure that they are not specific to the questionnaire we used, we conducted a replication with 647 new participants. The design and the analyses were exactly as in the main experiment. The only difference was that instead of using MPIQ in the main experiment, in the replication experiment, we used the first 10 items of the widely used BPS [44].

The full results of the experiment are reported in electronic supplementary material, figures S2−S4 and table S3. Overall, the results were similar to those reported in the main text, while the ‘No requirements’ and ‘95% approval’ participants, in contrast to ‘Master workers’ participants, showed many errors in ‘catch’ trials (electronic supplementary material, figure S2), exhibiting low reliability (electronic supplementary material, figure S3) and low inter-trial variability (electronic supplementary material, figure S4). The only minor difference from the main dataset was that for the most stringent ‘catch’ criterion (i.e. ‘arithmetic and all common-sense catch trials’), the ‘No requirements’ and ‘95% approval’ cohorts exhibited slightly higher reliability (i.e. they were closer to, albeit still worse than ‘Master workers’; electronic supplementary material, figure S3, bottom-right) and showed inter-trial variability similar to the ‘Master workers’ cohort (electronic supplementary material, figure S4, bottom).

## 4. Discussion

In the present study, conducted with a main group of participants and replicated with a new group of participants, we systematically examined quality and reliability of ‘No requirements’, ‘95% approval’ and ‘Master workers’ cohorts of MTurk workers. We found that ‘Master workers’ rarely missed attentional checks, had relatively high variability between trials (i.e. no tendency toward straightlining), and exhibited high reliability. In contrast, both ‘No requirements’ and ‘95% approval’ cohorts missed many attentional checks, had relatively low variability between trials (i.e. a tendency toward straightlining) and exhibited low reliability. Below we discuss our results in detail.

### 4.1. Quality and reliability of master workers

Master workers comprise an elite cohort of workers selected by MTurk based on workers’ previous performance. Surprisingly, as our survey showed (figure 1), master workers have rarely been used in research, probably because this option has been less known and less often recommended. For example, Martin Hebart in his online MTurk guide [50] suggested that master workers tend to be better than non-master workers, but the difference was not major. Two additional studies also did not report added value in using master workers [21,23], although in one of them [21] the master and non-master groups were not compared directly (i.e. statistically), and another study [23] used internal consistency Cronbach’s α to measure reliability, a potentially biased measure (see §1). Notably, as the data quality of MTurk changes, the conclusions obtained several years ago might not hold. Here, we show that master workers resulted in incomparably better data quality than workers without master qualifications. Specifically, master workers very rarely missed attentional checks. Master workers exhibited relatively high variability across trials (i.e. no tendency towards straightlining) and demonstrated high reliability calculated as within-subject across two sets of repeated items correlation. From a demographic point of view, master workers were older and more evenly distributed with regard to age compared with workers without master requirement. The gender proportion of the master workers group was also more balanced compared with the workers without master qualifications. For recruiting master workers, MTurk charges additional fees of 5% of the participant’s reward. Given that the standard MTurk fee rate stands at 40% of the participant’s reward (for batches of more than nine participants), paying only an additional 5% for high-quality workers might be worthwhile. So, from a data quality point of view, master workers seem to be an excellent solution. However, two potential limitations of using master workers should be taken into account. First, master participants are experienced or even very experienced participants, and therefore might not be as naive when performing the experiments. Similar concerns about over-experienced participants have been raised previously regarding MTurk workers in general [6], although it is not clear to what extent prior extensive experience biases the results of any type of experiment. It is plausible that a participant who repeatedly took part in experiments involving deception [51] or learning through incentives [52] will be biased when performing a similar new experiment. However, when participants are filling out a questionnaire on everyday mobile use or boredom, as was the case in our study, they only need to provide sincere answers, so prior experiment participation might not be a major biasing factor. Second, the size of the MTurk pool of available master workers is unclear. For our study (both the main and replication experiments), we recruited 161 master participants, but we do not know the extent to which the pool of potential participants is larger than this. Taken together, if the naivety of participants is not a strong prerequisite and a large sample size is not required, it is worthwhile to consider using master participants.

### 4.2. Quality and reliability of workers without master qualifications

The two other cohorts tested in the present study were ‘No requirements’ and ‘95% approval’ workers. Note that both these cohorts could potentially include master workers, although the proportion of master workers was probably very small, if present at all; otherwise, the results for these cohorts would have been similar to those of master workers. We found that both cohorts of participants without master requirement missed a large percentage of attentional catches, while less than 20% of the participants answered all five attentional catch items correctly. To this extent, we have serious doubts whether we can trust the responses of participants who respond that a runner would not be tired after completing a marathon in extremely hot weather or that a person would be glad to be diagnosed with cancer. The participants without master requirement also exhibited low reliability and low variability across trials. Low variability across trials indicates the so-called straightlining tendency [25,26], characteristic of careless participants who do not sufficiently invest in their tasks. In general, when workers were excluded based on ‘only arithmetic catches’ or ‘arithmetic and intuitive common-sense catches’, the remaining data were of very poor quality. When workers were excluded using the most stringent ‘arithmetic and all common-sense catches’ exclusion criterion, the remaining data were of better quality, particularly in the replication experiment, but still worse than the master participants’ data quality. The higher reliability and greater variability across trials observed in the replication experiment, compared with the main experiment, may be related to the fact that only the replication experiment included reverse-coded items (3 out of 10). Notably, applying the ‘arithmetic and all common-sense checks’ criterion requires exclusion of more than 80% of workers—the procedure which might not be practical. On the one hand, if the excluded participants are paid at the end of the experiment, this means that the researcher needs a budget six times larger for the study. On the other hand, if the excluded participants are not paid at the end of the experiment (i.e. their jobs are rejected), this will seriously lower the approval rating of the requester (i.e. the researcher), potentially causing difficulties in recruiting participants (i.e. the workers might tend to avoid requesters who approve a low percentage of their jobs). Overall, running experiments with workers without applying requirements or with a 95% approval rate—the two most frequently used options today (figure 1)—might be problematic and challenging.

### 4.3. Deteriorating quality of Mechanical Turk data

Initially, 10–15 years ago, the quality of MTurk data was excellent. For example, an influential study by Peer *et al*. [12] demonstrated that high-quality data can be achieved by recruiting workers with an approval rate of 95% or higher, even without the need for attentional checks. Over time, concerns about MTurk data quality were raised [5–8], and our present results fully confirm those concerns. We found that the data quality using a 95% approval threshold was practically no better than that of workers without requirements, while in both cohorts the quality was relatively low. Our finding that there is no difference between workers recruited without any restrictions and those with a 95% approval rate is in line with the result of Robinson *et al*. [19], who found that almost all MTurk workers recruited without restrictions had an approval rate of at least 95%, possibly due to the fact that requesters rarely reject jobs [10]. Interestingly, back then in 2019, Robinson *et al*. [19] reported that the data quality among both groups—those recruited without restrictions and those with a 95% approval-rate threshold—was high, but, as our results show, this is no longer the case today. Taken together, our results emphasize that the earlier recommendations for a 95% approval rate [12,15,16] are obsolete and no longer meaningful. It is possible that using a higher approval-rate criterion, possibly complemented by a minimum number of completed tasks, may improve data quality. For example, recruiting participants with an approval rate of 99.6% or higher—the average approval rate of Master workers in our study—may result in better data quality. However, instead of searching for the optimal parameters of approval rate and minimum number of completed tasks, it is simpler to use Master workers, who have already been selected by MTurk based on their previous performance.

### 4.4. Insights on using attentional checks

Our study provides two insights into attentional catches and their use. First, the arithmetic ‘catch’ trials were inefficient, as even mediocre participants without master qualification missed only a few arithmetic attentional catches. The likely reason for this was that arithmetic catch items were visually very distinct from other items; in other words, they ‘popped up’ (e.g. arithmetic catch items consisted of numbers instead of text, and the arithmetic catch item’s text was also shorter). Also, excluding participants who missed arithmetic attentional catches did not improve reliability (figure 2 and electronic supplementary material, figure S3). Taken together, attentional catches that are visually distinct are probably useless and better avoided. The second insight relates to the core assumption of attentional catch usage, namely that discarding participants who fail with attentional catches improves data quality, possibly substantially. In our study, the attentional catches formulated intuitively were missed by approximately half the participants, but discarding these participants only modestly, and in unsatisfactory way, increased reliability. For example, in the main dataset, ‘No requirements’ cohort, reliability increased from 0.11 to 0.16, while the reliability of ‘Master workers’ cohort was above 0.7. Note that in a typical study, reliability usually cannot be tested, as it requires a special design, as in our study. So researchers might assume that excluding participants who missed attentional catches, especially using stringent criteria, results in a substantial improvement in data quality. However, we show that this is not necessarily the case, and discarding participants who missed the attentional catches should not be considered a panacea for improving data quality.

### 4.5. The approach used to estimate participants’ reliability

To estimate reliability, in the present study, we calculated within-subject correlations across original and rephrased items [34,35]. The assumption behind this method is that attentive and diligent participants are expected to give similar responses to two questions with the same content, thus resulting in relatively high correlations between the two series of items. This method is particularly suitable because it specifically evaluates the reliability of participants. Similar method of validating participants’ responses to two items with similar content has been adopted in many inventories, including the Variable Response Inconsistency and True Response Inconsistency scales in the MMPI [36], the Millon Clinical Multiaxial Inventory [53] and the California Personality Inventory [54]. As we explained in detail in §1, the use of Cronbach’s α internal consistency to evaluate participant reliability might not be suitable because: (i) it measures the reliability of the questionnaire, not of the participant; and (ii) it can be biased when the quality of data is low, as in the case of straightlining response patterns [30,32,33]—as was the case in our data. To this end, methods like per-item correlation, mean-item correlation or total score agreement may also be suboptimal or even unsuitable for evaluating participant reliability for similar reasons. That is, on the one hand, per-item correlation and mean-item correlation are used to assess the quality of the questionnaire, but not the quality of the participants. On the other hand, results from analyses at the questionnaire-average level using across-subjects correlations (e.g. mean-item correlation, total score agreement methods) may be susceptible to straightlining and random response patterns [55]. For example, in an extreme scenario, if each participant provides random responses within a specific range (e.g. participants 1−10 respond randomly within the range 1–3, participants 11−20 within 2–4 and participants 21−30 within 3–5), the mean-item correlation between the two sets of items, calculated across participants, could be very high—even though the data are entirely random. Overall, the approach we used—calculating within-subject correlations across original and rephrased items—best addressed the goals of the present study.

### 4.6. Potential future directions

In the present study, conducted with more than 1300 participants across three cohorts, we used two questionnaires and three types of ‘catch’ questions. Despite the comprehensive nature of the study, some questions remain open and may warrant future investigation. First, our questionnaires included 16 (main experiment) and 20 (replication experiment) questions of interest, along with five ‘catch’ questions. Thus, the experiment was relatively short and contained a relatively high percentage of ‘catch’ questions—both factors that may have contributed to increased participant vigilance. In the future, it may be interesting to test data quality in studies conducted with longer questionnaires and lower percentage of ‘catch’ questions. Second, the present study focused specifically on examining data quality on the online MTurk platform. However, it may also be valuable to compare the quality of questionnaire data collected online with that collected offline (i.e. in the laboratory). Finally, MTurk workers differ significantly from college students—the cohort commonly used in many psychological studies. For example, while the average age of college students is around 20, the average age of participants across all cohorts in our study was above 30, and for ‘Master workers’ it was above 40. Therefore, in future work, it may be interesting to compare the data quality of participants from online platforms like MTurk with that of college student samples.

### 4.7. Conclusions and implications

In the present study, using main and replication experiments conducted with more than 1300 participants, we systematically investigated the data quality of MTurk workers. We found that the data quality of master workers is excellent, and therefore we recommend using them, particularly when the naivety of participants is not a strong prerequisite and a large sample size is not required. In contrast, the quality of workers without a master requirement, even when requiring a 95% or above approval rate, was poor and problematic for use. Finally, it should be emphasized that the goal of the present study was to evaluate the data quality of MTurk data, which is especially relevant for many researchers who continue using MTurk in their research. But as it was suggested previously [10,18,48], it is possible that other solutions result in better data quality and may be preferred over MTurk.

## Data Availability

Data and code are provided in the supplementary material [56].
